# Skeletal muscle single fiber force production declines early in juvenile male mice with chronic kidney disease

**DOI:** 10.14814/phy2.15651

**Published:** 2023-04-05

**Authors:** Brent A. Momb, Edwin Patino, Oleh M. Akchurin, Mark S. Miller

**Affiliations:** ^1^ Department of Kinesiology University of Massachusetts Amherst Massachusetts USA; ^2^ Joan and Sanford I. Weill Department of Medicine, Division of Nephrology and Hypertension Weill Cornell Medicine New York New York USA; ^3^ Department of Pediatrics, Division of Pediatric Nephrology Weill Cornell Medicine New York New York USA; ^4^ New York‐Presbyterian Hospital New York New York USA

**Keywords:** chronic kidney disease, cross‐bridge, myosin, pediatric, skeletal muscle

## Abstract

Children with chronic kidney disease (CKD) frequently exhibit delayed physical development and reduced physical performance, presumably due to skeletal muscle dysfunction. However, the cellular and molecular basis of skeletal muscle impairment in juvenile CKD remains poorly understood. Cellular (single fiber) and molecular (myosin‐actin interactions and myofilament properties) function was examined ex vivo in slow (soleus) and fast (extensor digitorum longus) contracting muscles of juvenile male (6 weeks old) CKD and control mice. CKD was induced by 0.2% adenine diet for 3 weeks starting at 3 weeks of age. Specific tension (maximal isometric force divided by cross‐sectional area) was reduced in larger myosin heavy chain (MHC) I and IIA fibers and in all IIB fibers in juvenile male mice with CKD due to fewer strongly bound myosin‐actin cross‐bridges. Fiber cross‐sectional area in juvenile CKD mice was unchanged in MHC I and IIB fibers and increased in MHC IIA fibers, compared to controls. CKD slowed cross‐bridge kinetics (slower rate of myosin force production and longer myosin attachment time, *t*_on_) in MHC IIA fibers, and accelerated kinetics (shorter *t*_on_) in MHC IIB fibers, which may indicate fiber type dependent shifts in contractile velocity in juvenile CKD. Overall, our findings show that single fiber myopathy is an early event during juvenile CKD, manifesting prior to the development of cellular atrophy as reduced force generation due to fewer strongly bound myosin heads. These results warrant clinical translation and call for early interventions to preserve physical function in children with CKD.

## Graphical abstract

Juvenile mice with early‐stage CKD display reduced single fiber contractile function. Cellular force production is lower primarily due to less strongly bound myosin‐actin cross‐bridges. Additionally, myosin‐actin cross‐bridge kinetics slow in myosin heavy chain IIA fibers.

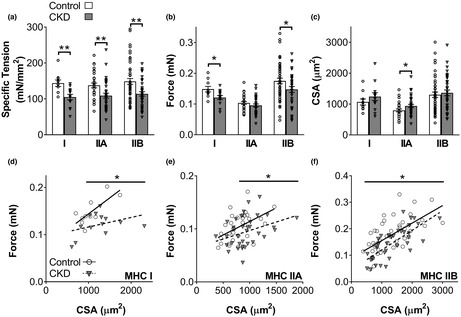

## 1 INTRODUCTION

Chronic kidney disease (CKD) is a term encompassing all individual progressive kidney disorders associated with a gradual loss of kidney function, which culminates at end‐stage kidney disease (ESKD) or kidney failure (Webster et al., 2017). The United States has one of the highest incidence rates of ESKD in the world (Thurlow et al., 2021). While the exact prevalence of CKD in children in the United States is unknown, conservative estimates indicate that between 200,000 and 300,000 children are affected (Saydah et al., 2018). CKD is a systemic disease that affects many organ systems with well recognized complications including anemia, mineral and bone disorder, metabolic acidosis, hypertension, and nutritional problems (Gerson et al., 2010). CKD in children is distinct from CKD in adults because of the different spectrum of primary diseases causing CKD and the unique clinical manifestations of CKD in children. Children with CKD frequently exhibit impairment of statural growth and delayed neurocognitive, physical, and sexual development (Foster et al., 2011; Gerson et al., 2010). Poor physical performance and limited motor function is also present in adults with CKD, especially in elderly patients, and can be associated with CKD‐related cachexia, including sarcopenia or decreased muscle mass (Hiraki et al., 2013). In children, CKD‐related myopathy is unique in that while uremic toxins and other metabolic alterations affect skeletal muscle similarly to adults, muscle tissue itself is still undergoing age‐dependent growth and development in children with CKD, although frequently with a delay compared to healthy children (Foster et al., 2011). Poor physical function correlates with progression of pediatric chronic kidney disease (Baek et al., 2017; Gerson et al., 2010) and is a particularly strong determinant of the suboptimal quality of life of children with CKD, which may impact their social adaptation later in life (Foster et al., 2011; Gerson et al., 2010). However, the mechanisms behind loss of physical performance in children with CKD are incompletely understood, which is one of the barriers to create effective interventions.

Proposed putative explanations of whole‐muscle function decline in pediatric patients (Foster et al., 2011; Hogan et al., 2020) include loss of nervous system activation (neuropathy), skeletal muscle mass (atrophy) and/or skeletal muscle intrinsic contractile capacity (myopathy), all of which have been also found to some extent in adults with CKD (de Souza et al., 2017; Diesel et al., 1990; Fahal et al., 1997; John et al., 2013; Krishnan & Kiernan, 2007; Petersen et al., 2012; Zhou et al., 2018). Determining the individual impact of each of these components using whole body and whole muscle function can be difficult, so a reductionist approach may be needed to determine the specific mechanisms behind the loss of physical capacity. One means of examining myopathy independent of neuropathy and other confounders is to examine single fiber contractile function as these measurements are performed on fibers without an excitable membrane, due to being chemically skinned, and within their normal three‐dimensional structure, protein content, and ionic conditions (Höök et al., 2001; Miller et al., 2013; Miller et al., 2015; Mitrou et al., 2019; Momb et al., 2022; Toth et al., 2013). Single fiber force production (cellular level) and the underlying myofilament mechanical properties (molecular level), including myosin‐actin cross‐bridge kinetics, can be assessed within the same fiber (Kawai & Halvorson, 1991; Miller et al., 2010; Momb et al., 2022; Zhao & Kawai, 1994). To our knowledge, no human studies have examined cellular and molecular contractile function in children with CKD, potentially due to the difficulties of performing skeletal muscle biopsies in this population. However, animal models have been developed and used to examine the effects of CKD on a variety of physiological systems. Using an adult murine model of CKD, we and others have demonstrated that single skeletal muscle fiber force production was reduced as evidenced by the 28–51% lower specific tension (maximal isometric force divided by cross‐sectional area) (Mitrou et al., 2019; Momb et al., 2022) in each of the four fiber types (myosin heavy chain or MHC I, IIA, IIX, and IIB). Our recent work shows this loss of force production is due to a reduced number of strongly bound myosin‐actin cross‐bridges and/or decreased myofilament stiffness in adult CKD mice (Momb et al., 2022). Additionally, slower myosin‐actin cross‐bridge kinetics were found with CKD in MHC I and IIA fibers, which may potentially influence contractile velocity (Momb et al., 2022).

These prior studies using animal models (Mitrou et al., 2019; Momb et al., 2022) have provided initial insights into the underlying contractile deficits of advanced CKD in adults as, to our knowledge, no human studies have reported these measures. Furthermore, no human or animal studies have examined the impacts of early‐stage CKD on cellular and molecular contractile function. Given the differences between juvenile and adult CKD (Baek et al., 2017; Becherucci et al., 2016), elucidating whether juvenile CKD has any distinct effects on skeletal muscle is critical. Thus, the purpose of this study was to determine early alterations of skeletal muscle fiber contractile function in juvenile male mice with CKD.

## 2 METHODS

### 2.1 Animals and experimental design

Protocols, as previously described (Akchurin et al., 2016), were approved by the Institutional Animal Care and Use Committee of Weill Cornell Medicine and complied with the National Research Council *Guide for the Care and Use of Laboratory Animals*. Male C56BL/6J mice purchased from Jackson Laboratories at 3 weeks of age were randomly assigned to control (*N* = 6) or CKD (*N* = 5) groups. Mice were housed in standard cages at ~22°C on a 12 h light–dark cycle with ad‐libitum food and water intake. CKD was induced by a 0.2% adenine diet for 3 weeks. We have previously validated the use of the adenine diet in juvenile male mice indicating that various phenotypes are similar to humans including growth retardation, cachexia, anemia, iron deficiency and inflammation (Akchurin et al., 2016). Both groups received intraperitoneal injections of iron dextran at 0.5 g/kg weekly to eliminate the potential confounding effect of iron deficiency and anemia on muscle contractile function (Dziegala et al., 2018; Finch et al., 1976; Momb et al., 2022; Patino et al., 2020). Mouse body length was measured by the nose (snout) to tail tip length of anesthetized mice. CKD was initiated at 3 weeks and the mice were euthanized via cervical dislocation after isoflurane anesthetization at 6 weeks of age, which corresponds to approximately 4 years of CKD from 8 to 12 years old in human age (Dutta & Sengupta, 2016). The soleus and extensor digitorum longus (EDL) muscles, kidneys, as well as blood from the inferior vena cava were collected at euthanasia.

### 2.2 Kidney histology and function

Mouse kidneys were fixed in 4% paraformaldehyde (Santa Cruz Biotechnology) overnight and embedded in paraffin. Paraffin blocks were cut into 5 μm thick sections which were placed on positively charged slides, deparaffinized and stained with Masson's trichrome to assess fibrosis. Kidney fibrosis was quantified as percent of mid‐sagittal kidney sections affected by interstitial fibrosis (Sethi et al., 2017). Blood urea nitrogen (BUN) was measured on the Beckman Coulter AU 680 analyzer. Hemoglobin and red blood cell counts were measured on the IDEXX Procyte DX analyzer.

### 2.3 Single fiber measurements

Dissection, skinning and storage solutions for muscle tissue acquisition and storage as well as relaxing, preactivating and activating solutions for mechanical analyses were identical to those previously described (Momb et al., 2022). The preparation of single fibers and the experimental device used for measurement of specific tension and sinusoidal analysis has also been previously described (Momb et al., 2022). Briefly, approximately 1 mm long single fibers were isolated from soleus or EDL muscles, demembranated and mounted onto hooks attached to a piezo actuator linear motor (P‐841.10, Physik Instrumente) and an Akers strain gauge (AE‐801, SensorOne). Sarcomere length was set to 2.65 μm and elliptical cross‐sectional area (CSA) was calculated based upon measurements of top and side diameters using an inverted microscope (Zeiss Invertiscope) with a video camera (BFLY‐U3‐23S6m‐C, Point Grey Research) and custom video analysis software (ImageJ) (Schneider et al., 2012). Prior to activation, the fiber was slackened completely, the force gauge zeroed, the fiber pulled back to its original length, allowed to equilibrate for 1 min, relaxed specific tension measured and transferred to pre‐activating solution for 30 s. At this point, fibers were placed into activating solution with specific tension measured at its plateau and sinusoidal analysis performed. Sinusoidal analysis was performed at 25°C under maximal Ca^2+^‐activated conditions, which yields three characteristic processes, A, B, and C, that relate to various mechanical (*A*, *B*, *C* and *k*) and kinetic (2π*b* and 2π*c*) properties of the cross‐bridge cycle, as previously described (Momb et al., 2022). The A‐process (*A* and *k*) under Ca^2+^‐activated conditions reflects the myofilament lattice stiffness and attached myosin heads in series (Mulieri et al., 2002). The parameter *A* indicates the magnitude of the viscoelastic modulus, whereas *k* represents the degree to which these viscoelastic magnitudes are purely elastic (*k* = 0) or purely viscous (*k* = 1). The B‐ and C‐process magnitudes (*B* and *C*) are proportional to the number of strongly bound myosin‐actin heads and/or the stiffness of the cross‐bridges (Palmer et al., 2007). The frequency portion of the B‐process (2π*b*) is interpreted as the rate of myosin transition from the weakly to strongly bound state, or the (apparent) rate of myosin force production (Zhao & Kawai, 1993). The frequency portion of the C‐process (2π*c*) represents the cross‐bridge detachment rate, meaning the inverse (2π*c*)^−1^ is the mean myosin attachment time to actin, *t*_on_ (Palmer et al., 2007). Single fiber mechanical experiments were completed within 3 weeks of initial dissection.

### 2.4 Myosin heavy chain isoform composition

Following mechanical measurements, individual fibers were placed in 30 μL loading buffer, heated for 2 min at 65°C and stored at −80°C until determination of MHC isoform composition by sodium dodecyl sulfate‐polyacrylamide gel electrophoresis (SDS‐PAGE) to identify fiber type, as described (Miller et al., 2010), with the following minor modifications to achieve better separation between the four MHC isoforms expressed in mouse skeletal muscle: the resolving gel was comprised of 8% acrylamide/bis‐30% glycerol (weight/volume) and gels were run at 70 V for 1 h followed by 275 V for 26 h.

### 2.5 Statistical analysis

Data are expressed as mean ± SEM. For each fiber type, a linear mixed model was used to compare mean differences between control and CKD, including a random effect to account for multiple measurements within mice. Linear regression was conducted to examine relationships between force and other variables, such as CSA, the number of bound myosin heads, and/or their stiffness. ANCOVA was used to determine differences between slopes and y‐intercepts for regression lines. If differences in slope and intercept where found, moderation analysis using the Johnson‐Newman technique was performed to determine where differences occurred. Statistical significance was set at *p* ≤ 0.05 and analyses done using SPSS for Windows version 28.0 (IBM, Armonk, NY).

## 3 RESULTS

### 3.1 Chronic kidney disease characteristics

**FIGURE 1:**
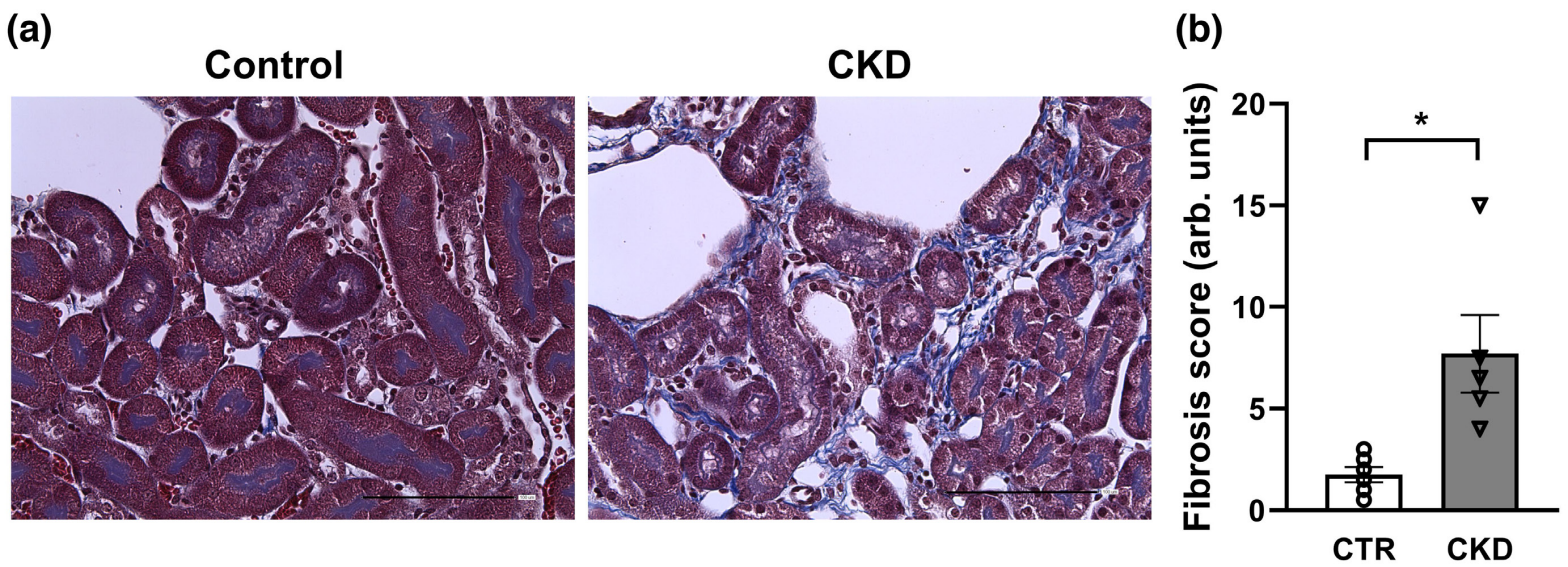
Kidney histology in control (CTR) and chronic kidney disease (CKD) juvenile male mice. (a) Masson's trichrome staining of kidney sections, representative images; ×40 magnification. Scale bars, 100 μm. (b) Quantification of kidney fibrosis. Mean ± SE are shown with individual mouse values overlaid onto each bar. *p ≤ 0.05.

Mice with CKD (*N* = 6) had slower weight gain and a trend (*p* ≤ 0.1) toward slower gain of body length compared to control (*N* = 5) mice (Table 1). Kidney weight, red blood cell counts, and hemoglobin were all similar between control and CKD mice at the end of experimental period (Table 1). CKD mice had a similar blood urea nitrogen and serum creatinine at the end of experimental period, indicating that mice remained at an early stage of CKD at the time of skeletal muscle retrieval (Table 1). However, kidney histology revealed development of fibrosis in juvenile male mice after 3 weeks of adenine diet (Figure 1). Of note, we have previously reported other systemic manifestations of CKD, such as mineral and bone disorder at that stage in the same juvenile model, despite no significant elevation of blood urea nitrogen and serum creatinine (Patino et al., 2020).

**TABLE 1:** Disease characteristics for control and chronic kidney disease (CKD) mice.

	Control (*n* = 6)	CKD (*n* = 5)	*p*‐value
Morphometric parameters			
Body weight at euthanasia (g)	18.6 ± 1.2	16.6 ± 1.5	0.31
Weight gain (%)	76 ± 9.8	41 ± 12.4	0.05
Body length at euthanasia (cm)	16.8 ± 0.3	16.5 ± 0.3	0.58
Length gain (%)	19 ± 1.7	13 ± 2.4	0.06
Left kidney weight (mg)	135.8 ± 10.4	128.4 ± 6.6	0.58
Right kidney weight (mg)	132.5 ± 9.7	132.2 ± 6.3	0.98
Hematologic and biochemical parameters			
RBC (10^6^/μL)	8.4 ± 0.2	9.0 ± 0.3	0.14
Hemoglobin (g/dL)	12.9 ± 0.5	13.7 ± 0.5	0.30
BUN (mg/dL)^a^	32.9 ± 3.1	37.5 ± 3.3	0.35
Creatinine (mg/dL)^a^	0.18 ± 0.02	0.22 ± 0.02	0.33

*Note*: Data are presented as mean ± SE.

CKD mice received adenine diet for 3 weeks beginning at 3 weeks of age.

Abbreviations: BUN, blood urea nitrogen; RBC, red blood cell.

^a^ Taken from separate group of mice (*n* = 5 control, *n* = 5 CKD).

### 3.2 Single fiber measurements

**FIGURE 2:**
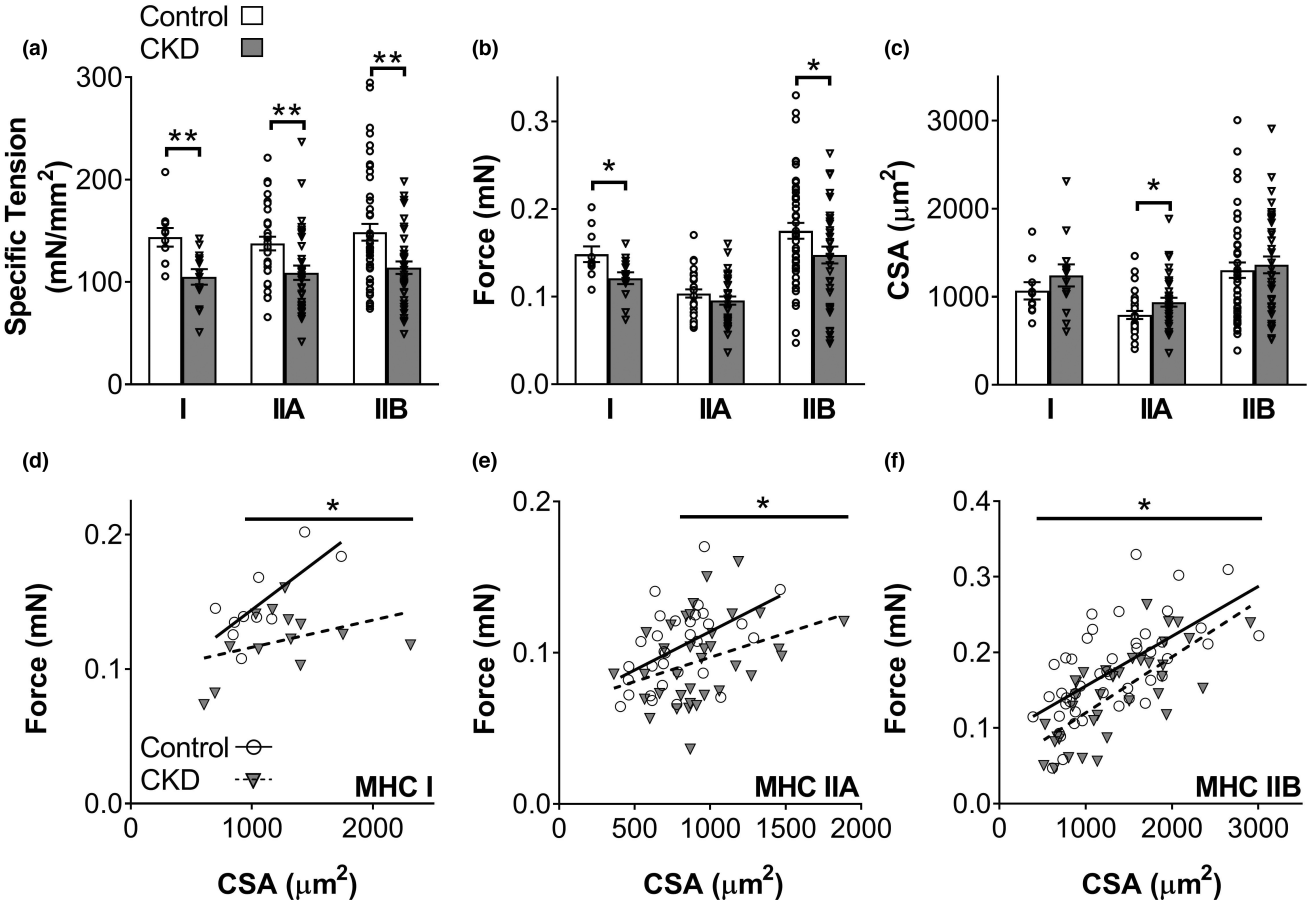
Single skeletal muscle fiber maximum Ca^2+^‐activated (pCa 4.5) specific tension, force, and cross‐sectional area (CSA) for control and chronic kidney disease (CKD) by myosin heavy chain (MHC) isoform (I, IIA, and IIB). Top (a‐c): Specific tension (force/CSA), absolute force, and CSA for control and CKD mice. Bottom (d‐f): Force versus CSA scatterplots, with each point representing an individual fiber, by MHC isoform. Solid and dashed lines indicate linear regression for controls and chronic kidney disease (CKD), with Pearson's correlation coefficients (*r*) for MHC I (0.74, *p* = 0.013 and 0.37, *p* = 0.208), IIA (0.49, *p* = 0.005 and 0.35, *p* = 0.041), and IIB (0.64, *p* < 0.001 and 0.73, *p* < 0.001) fibers. Intercepts were significantly different (*p* < 0.05) between control and CKD for MHC I and IIB fibers. Where significant differences in slope exist, regions of significance lines from moderation analysis are provided at the top of the scatterplot. Mean ± SE are shown with individual data points overlaid onto each bar. Number of fibers for control/CKD are as following, MHC I: 10/13, MHC IIA: 31/35, MHC IIB: 47/37. *p ≤ 0.05, **p ≤ 0.01.

*Force, CSA, and specific tension*: MHC I and IIA fibers were from the soleus and MHC IIB fibers were from the EDL. Mean maximal Ca^2+^‐activated isometric specific tension (single fiber force divided by CSA) in MHC I, IIA, and IIB fibers was lower in CKD compared to control (Figure 2a). Specific tension was lower in CKD due to reduced force production in MHC I and IIB fibers, as CSA remained similar, and due to increased CSA in MHC IIA fibers, as force production remained similar to control (Figure 2b,c). The force‐CSA relationships were examined for individual fibers and linear regression performed for both groups by MHC isoform. These plots show CKD fibers generate less force than control at large CSAs for MHC I (>950 μm) and IIA (>800 μm) fibers as indicated by moderation analysis (Figure 2d,e). For MHC IIB fibers, CKD had a lower intercept compared to control, showing force production was lower with disease across the entire range of CSAs (Figure 2f).

**FIGURE 3:**
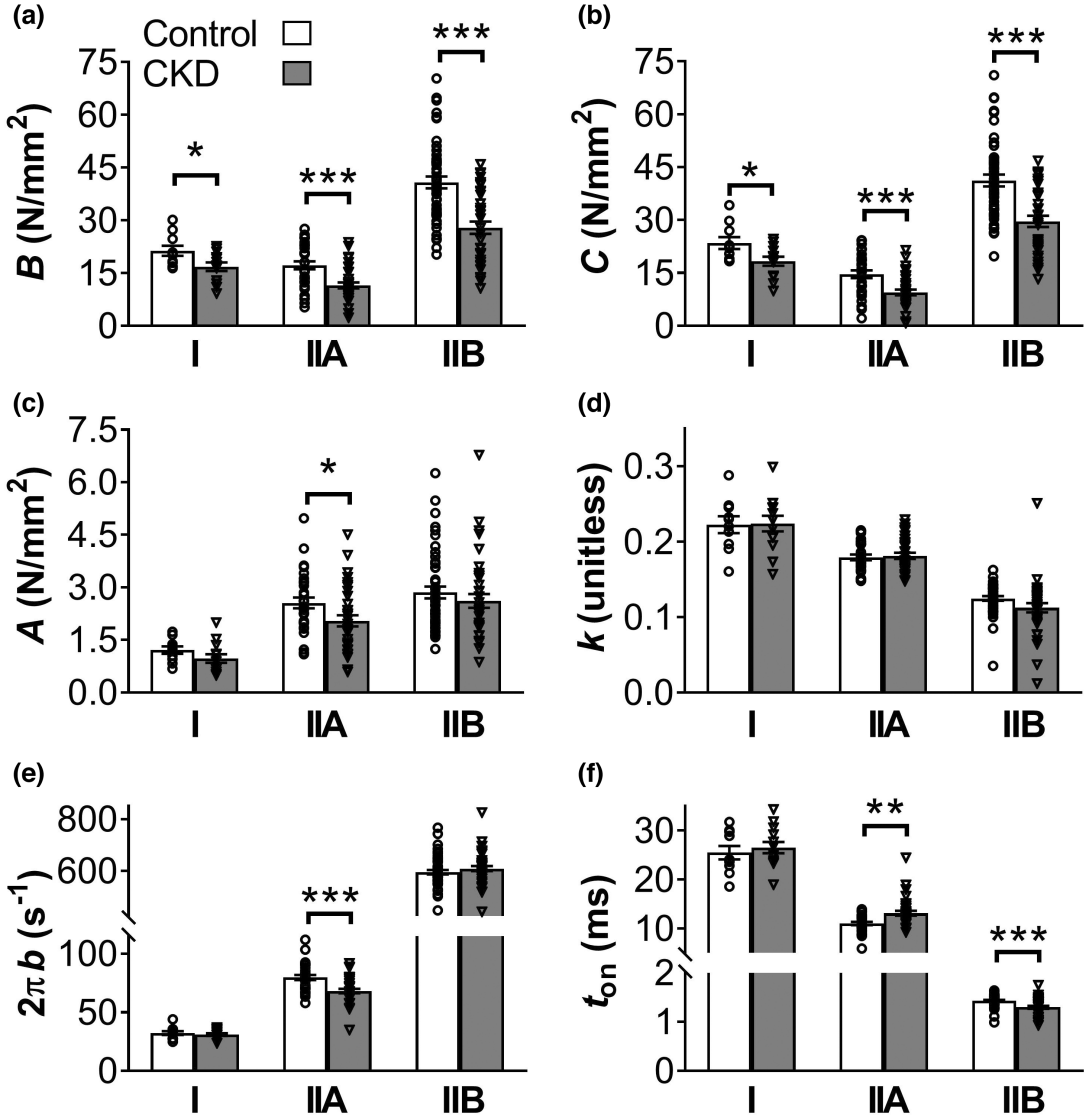
Mechanical parameters from sinusoidal analysis at maximum Ca^2+^‐activation (pCa 4.5) for control and chronic kidney disease (CKD) by myosin heavy chain (MHC) isoform (I, IIA, and IIB). (a, b) Parameters *B* and *C* are proportional to the number of myosin heads strongly bound to actin and cross‐bridge stiffness. (c, d) *A* and *k* represent the underlying stiffness of the lattice structure and the attached myosin heads. (e, f) 2*πb* and *t*_on_ represent myosin‐actin cross‐bridge kinetics where 2π*b* is the rate of myosin transition between the weakly and strongly bound states and *t*_on_ represents the mean time myosin is attached to actin. Mean ± SE are shown with individual datapoints overlaid onto each bar. Number of fibers for control/CKD are as following, MHC I: 10/13, MHC IIA: 31/35, MHC IIB: 47/37. *p ≤ 0.05, **p ≤ 0.01, ***p ≤ 0.001.

*Myofilament mechanics*: To evaluate the myofilament mechanics causing differences in specific tension between CKD and control, the six model parameters estimated from sinusoidal analysis during maximal Ca^2+^‐activation were examined. Parameters *B* and *C*, related to the number of myosin heads strongly bound and/or their stiffness per muscle size, were smaller in CKD in all fiber types (Figure 3a,b). The large reduction in *B* and *C* is likely caused by a decrease in the number of strongly bound cross‐bridges as recent modeling results indicate cross‐bridge stiffness only modestly alters isometric force production (Fenwick et al., 2017). The underlying stiffness of the lattice structure and the attached myosin heads per muscle size remained unchanged (no change in *A* or *k*) in MHC I and IIB fibers but decreased in magnitude (smaller *A*) in MHC IIA fibers (Figure 3c,d). The myosin‐actin cross‐bridge kinetics responded to CKD differently based upon fiber type (Figure 3e,f). In MHC I fibers, cross‐bridge kinetics were unchanged. In MHC IIA fibers, the rate of myosin transition between weakly and strongly bound states (2π*b*) became slower and myosin attachment time (*t*_on_) longer in CKD, showing an overall slowing of cross‐bridge kinetics. MHC IIB fibers displayed faster cross‐bridge kinetics with CKD as evidenced by a shorter *t*_on_.

### 3.3 Relationships between force and number of strongly bound myosin heads

**FIGURE 4:**
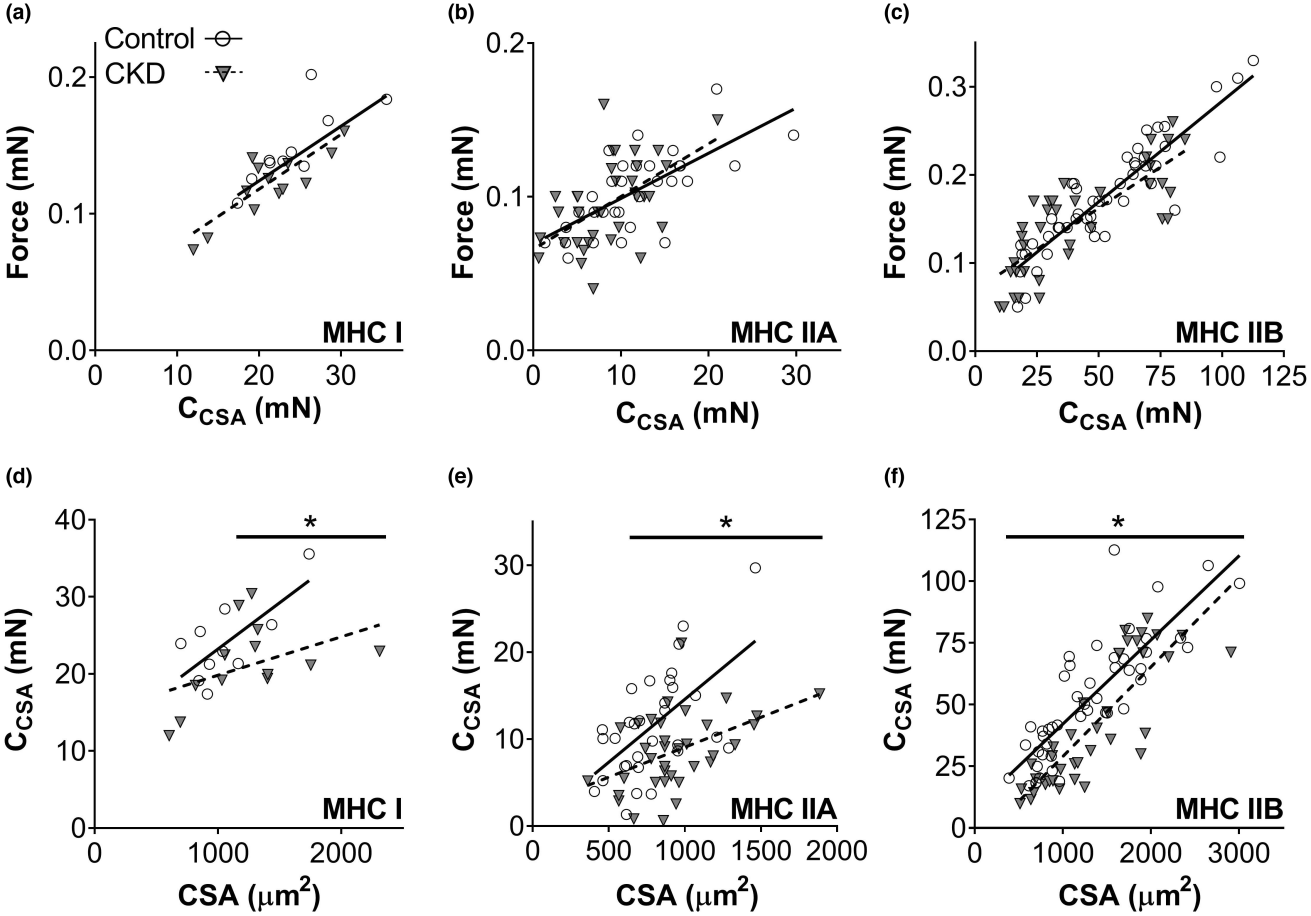
Relationship between force‐C_CSA_ and C_CSA_‐CSA for control and chronic kidney disease (CKD) by myosin heavy chain (MHC) isoform (I, IIA, and IIB). Data points represent results from individual fibers. Solid and dashed lines indicate linear regression for control or chronic kidney disease (CKD). a‐c: Pearson's correlations coefficients for force‐CSA in control and CKD for MHC I (*r* = 0.79, *p* = 0.006 and *r* = 0.84, *p* < 0.001), IIA (*r* = 0.70, *p* < 0.001 and *r* = 0.54, *p* = 0.001) and IIB (*r* = 0.90, *p* < 0.001 and *r* = 0.80, *p* < 0.001) fibers. Slopes and intercepts were similar for controls and CKD for all fiber types. d‐f: Pearson's correlations coefficients for C_CSA_‐CSA for control and CKD in fiber types MHC I (*r* = 0.72, *p* = 0.018 and *r* = 0.45, *p* = 0.120), IIA (*r* = 0.46, *p* = 0.005 and *r* = 0.60, *p* = 0.001), and IIB (*r* = 0.84, *p* < 0.001 and *r* = 0.83, *p* < 0.001) fibers. Intercepts were significantly different (*p* < 0.05) between control and CKD for MHC IIA and IIB fibers. Where significant differences between slope exist, regions of difference between control and CKD are shown by horizontal lines above the scatterplot. Number of fibers for control/CKD are as following, MHC I: 10/13, MHC IIA: 31/35, MHC IIB: 47/37. *p ≤ 0.05.

To understand the mechanisms behind alterations in force production, we examined the relationship between force and the number of strongly bound myosin heads (C_CSA_), or parameter *C* not normalized to CSA. This approach removes potential collinearity between force production and *C* [i.e., CSA is a well‐known predictor of force production] (Slinker & Glantz, 1985). Force is expected to increase with a greater number of strongly bound myosin heads (Miller et al., 2015). Indeed, force increased and was well correlated with C_CSA_ in all fiber types examined (*r* = 0.54–0.90, *p* = 0.006 to ≤0.001) (Figure 4a–c). However, the slopes and intercepts were similar between groups for all fiber types, indicating force was similar in CKD and controls when the same number of myosin heads were strongly bound.

The relationships between C_CSA_ and CSA were examined to determine how the number of strongly bound heads were affected by fiber size. The C_CSA_‐CSA plots indicate CKD fibers have fewer strongly bound myosin heads at larger CSAs in MHC I (>1100 μm) and IIA (>650 μm) fibers as indicated by a reduced slope in CKD and examination of the differences in regression lines (Figure 4d,e). At all CSAs for MHC IIB fibers, the number of strongly bound myosin heads were smaller in CKD, as indicated by their lower intercept but similar slope to controls (Figure 3f). The similarity in the relationships between force and CSA (Figure 2d–f) as well as force and C_CSA_ (Figure 4d–f) further supports our finding that CKD caused reduced force production from a reduction in the number of strongly bound myosin heads.

## 4 DISCUSSION

Our findings show that chronic kidney disease (CKD) alters skeletal muscle function at the cellular and molecular levels in juveniles early in the disease course, before the development of muscle atrophy (sarcopenia). Importantly, we examined both slow (soleus) and fast (extensor digitorum longus) contracting muscles to cover numerous fiber types, including the most common fiber types in human skeletal muscle, MHC I and IIA (Galpin et al., 2012; Luden et al., 2008; Miller et al., 2013). We found that force production was reduced by CKD in all fiber types examined (MHC I, IIA and IIB) and was associated with a decrease in the number of myosin heads strongly bound to actin. Myosin‐actin cross‐bridge kinetics responded to CKD differently based upon fiber type, with MHC I unchanged, MHC IIA slower, and MHC IIB faster than in healthy mice. Our study reveals very early alterations of skeletal muscle function at the molecular level in the course of juvenile CKD and suggests that they are at least partially responsible for reduced whole muscle isometric torque (Hogan et al., 2020) as well as decreased physical performance and frailty in children with CKD (Sgambat et al., 2019).

Specific tension dropped 21–30% after mild kidney impairment in CKD compared to control mice indicating that large changes in contractile function occur in juveniles with early‐stage CKD. These findings could lead to the impairment of ongoing musculoskeletal maturation, a well‐known complication of juvenile early‐stage CKD. Our recent work in adult CKD mice showed a larger loss in specific tension (36%–51%), but this was after eight weeks of adenine diet (Momb et al., 2022), more advanced CKD than in the current study. Contractile characteristics that can explain the mechanisms behind reduced specific tension were similar between juvenile early‐stage and adult late‐stage CKD, but the magnitude of changes was typically greater in adult late‐stage CKD (Table 2). In both early‐ and late‐stage CKD, the primary mechanism behind reduced specific tension is likely a loss of strongly bound myosin‐actin cross‐bridges. Secondarily, a small drop in myofilament stiffness in MHC IIA fibers occurs in juvenile early‐stage CKD, whereas a large drop in myofilament stiffness is occurring in adult late‐stage CKD MHC IIA and IIB fibers. While cross‐bridge kinetics begin to slow in MHC IIA fibers in juvenile early‐stage CKD, slowing of kinetics impacts both MHC I and IIA fibers in adult late‐stage CKD. As slowing of cross‐bridge kinetics, especially longer myosin attachment times (*t*_on_), can lead to slower single fiber contractile velocities (Brizendine et al., 2017; Miller & Toth, 2013), if changes in velocity occur with disease progression in CKD, they may be expected to be fiber type dependent (Table 2) and should be examined in future work.

**TABLE 2:** Functional changes in mouse and human single skeletal muscle fibers with chronic disease (chronic kidney disease, heart failure, and cancer) and during aging.

Variable	MHC Isoform	Mice			Humans		
		Juvenile Early‐CKD	Adult Late‐CKD (Momb et al., 2022)	Δ (%)	Heart Failure (Miller et al., 2010)	Cancer (Toth et al., 2013)	Aging (Miller et al., 2013)
Cellular							
Specific tension	I	**↓**	**↓↓**	12	**↔**	**↔**	**↔↑**
	IIA	**↓**	**↓↓**	18	**↔**	**↓**	**↔↑**
	IIB	**↓**	**↓↓↓**	28			
Contractile velocity^a^	I	↔	**↓**		**↓**	**↓**	**↓**
	IIA	**↓**	**↓↓**		**↓**	**↔**	**↓**
	IIB	↑	↔				
Molecular							
Strongly bound cross‐bridges	I	**↓**	**↓↓↓**	46	**↔**	**↔**	**↔**
	IIA	**↓↓**	**↓↓**	15	**↔**	**↓**	**↔**
	IIB	**↓↓**	**↓↓**	11			
Myofilament lattice stiffness	I	↔	↔	5	**↑↑**	**↔**	**↑**
	IIA	**↓**	**↓↓**	28	**↑**	**↔**	**↑**
	IIB	↔	**↓↓↓**	44			
Cross‐bridge kinetics	I	↔	**↓**	17	**↓**	**↓**	**↓**
	IIA	**↓**	**↓↓**	20	**↓**	**↔**	**↓**
	IIB	↑	↔	4			

Abbreviations: ↑, increase/faster. ↔, no change. ↓, decrease/slower; number of arrows signifies magnitude of response with one arrow indicating 10%–30% change, two arrows 30%–50%, and three arrows >50%; two arrows in different directions in same cell (i.e., specific tension changes with aging) indicate male and female responses; Δ (%) is the absolute difference between the adult late‐chronic kidney disease (CKD) and juvenile early‐CKD response compared to their respective controls; cross‐bridge kinetics is the combined average change of 2π*b* and *t*_on_; MHC = myosin heavy chain; Humans do not express MHC IIB fibers, so there is no cellular or molecular data available for this fiber type.

^a^ Predicted from myosin attachment time differences.

Our study is the first to examine the effects of CKD in juvenile mice on single muscle contractile function and highlights the need for further longitudinal assessment of muscle dysfunction at various CKD stages and in different age groups. For instance, although the disease effects were smaller in juveniles from this study compared to adults (Momb et al., 2022), we are unable to determine if this is due to the age difference (juveniles vs. adults) and/or length of having CKD (early‐stage for juveniles vs. late‐stage for adults). Our study clearly shows for the first time that CKD affects single fiber function in juveniles, but whether the disease manifests differently in juveniles as their muscle tissue is in a different stage of growth and development compared to adults remains unknown.

Juvenile early‐stage CKD mice showed decreased contractile function at the very early stages of CKD, that could be detected by kidney histology, but not even by serum creatinine measurements. As skeletal muscle growth is physiologically very fast between 21 and 44 days of life for juvenile mice (White et al., 2010), loss of contractile function may be due to slow muscle protein accretion and/or increased protein breakdown, both found in CKD (Peng et al., 2016; Zhang et al., 2020), and consistent with slower weight gain in our CKD mice compared to controls. The loss of strongly bound cross‐bridges in MHC I, IIA and IIB fibers (Figure 4a,b) and reduced myofilament stiffness in MHC IIA fibers (Figure 4c) may be due to loss and/or degeneration of myofilaments and Z bands found in adult CKD patients on dialysis (Ahonen, 1980; Diesel et al., 1993; Lewis et al., 2012), but these findings have not yet been replicated in children with CKD. As CKD did not reduce fiber size in the present study, myosin loss could have occurred along the length or in the periphery of the myofilaments instead of radially. Our prior work in heart failure patients indicate myosin may be lost randomly along the thick filament, leading to reduced strongly bound cross‐bridges (Miller et al., 2009; Miller et al., 2010), and modeling indicates this could result in slower kinetics (Tanner et al., 2014). However, our finding of no loss in fiber size should be examined in more detail in future studies using additional techniques, such as immunohistochemistry, that can measure a larger number of fibers from an individual muscle. Overall, our findings suggest that initial events in CKD‐associated myopathy are likely functional and potentially reversible, with a potential for further structural alterations at the end of myofilaments. Further studies should build upon our work to examine upstream regulatory mechanisms responsible for slowed cross‐bridge kinetics or loss of strongly bound cross‐bridges that were observed.

Children with CKD have unique comorbidities compared to adults (Baek et al., 2017; Becherucci et al., 2016), and display phenotypical premature aging (Kooman et al., 2014) in part from sedentary lifestyles, increased allostatic load, and impairment of anti‐aging pathways that are also implicated in heart failure (Guidi et al., 2016; Pim van der et al., 2010) and cancer (Hurria et al., 2016). Comparing our previous human work examining aging (Miller et al., 2013), heart failure (Miller et al., 2010), and cancer (Toth et al., 2013) to our current animal work in CKD provides insights into similarities and differences in the skeletal muscle response to aging and disease (Table 2). These data are not representative of the entire literature for aging, heart failure, or cancer, but the use of the same methodological approach makes comparisons suitable and valuable across aging and disease. These comparisons are performed for MHC I and IIA fibers as humans do not express the MHC IIB isoform. Specific tension and the number of strongly bound cross‐bridges was decreased in CKD, but remained unchanged or increased in heart failure, cancer and aging, except for MHC IIA fibers from cancer patients. Myofilament lattice stiffness was primarily decreased in late‐stage CKD, unchanged in cancer and early‐stage CKD, but increased with heart failure and aging. Cross‐bridge kinetics tended to slow with aging and disease, leading to potential decreases in contractile velocity. Overall, while slower cross‐bridge kinetics appear to be common with aging and kidney‐unrelated chronic conditions, CKD‐related myopathy is unique because this disease results in lower force production compared to controls and is likely due to fewer strongly bound myosin‐actin cross‐bridges and, in some cases, reduced myofilament stiffness. Thus, the multifactorial deficits in molecular contractile function in CKD point to unique mechanisms behind myopathy not present in other conditions and highlight the importance of early kidney‐skeletal muscle crosstalk in juvenile CKD, which has recently emerged as a focus of intensive investigation (Solagna et al., 2021).

The strengths of this study include a well‐established and previously validated animal model of juvenile CKD, and the state‐of‐the art ex vivo assessment of live skeletal muscle function at the single fiber level. However, our study also had some limitations. While our results indicate that CKD does produce substantial changes in cellular and molecular function of skeletal muscle in juvenile male mice, the precise mechanism of these changes remains to be elucidated. Recently, kidney‐muscle crosstalk has received unprecedented attention in the literature (Leal et al., 2021). The changes that we observe could owe to the direct effect of the uremic toxins or mediators; however, there is potential for a reduction in physical activity or changes in eating patterns to affect our results. Previously, we examined muscle disuse in knee osteoarthritis patients and the results indicated that the cross‐bridge function and sacromeric structure did not change to the same extent as found in this model of CKD (Callahan et al., 2015; Miller et al., 1985). Similar to our findings, others have described large changes to myofilament structure in humans with advanced CKD, which is not typically seen with aging or disuse (Diesel et al., 1993; Lewis et al., 2012). Taken together, these findings suggest that while disuse and/or malnutrition may partially explain our results, the primary driver is likely reductions in kidney function. Another limitation to this study is our use of only male mice; however, as these mice were pre‐pubescent for the majority of the study period, changes in structure/function of skeletal muscle may not be impacted by hormonal influences near to the extent as adults would.

In conclusion, this work makes important steps toward unraveling the pathophysiology of skeletal muscle alterations in juvenile CKD. Notably, we observed early decreases in the number of myosin heads strongly bound to actin in MHC I, IIA and IIB fibers as well as fiber‐type specific changes in myosin‐actin cross‐bridge kinetics. These findings indicate that physical function in juvenile CKD is being impaired early in the disease course on the molecular level due to intrinsic contractile deficits independent of skeletal muscle atrophy. Thus, with timely interventions, these changes could potentially be reversible. Our findings set the stage for future studies to further delineate how disease progression uniquely affects muscle dysfunction in juvenile CKD and to identify systemic mediators of these changes, as well as effective therapeutic interventions toward mitigation of physical function decline in children with CKD.

## AUTHOR CONTRIBUTIONS

O.A. and E.P. developed, maintained, and provided tissues and data for the mouse model; B.A.M. and M.S.M. designed fiber experiments; B.A.M. performed and analyzed fiber experiments under the supervision of M.S.M.; B.A.M. and M.S.M. wrote the first manuscript draft; all authors contributed to data interpretation and manuscript revision.

## ETHICS STATEMENT

This study was approved by the Institutional Animal Care and Use Committee of Weill Cornell Medicine and complied with the National Research Council Guide for the Care and Use of Laboratory Animals.

## FUNDING INFORMATION

This work was supported in part by the National Institutes of Health, National Institute of Diabetes and Digestive and Kidney Diseases K08 DK114558 (OA), and Weill Cornell Medicine Rohr Family Clinical Scholar Award (OA).

## CONFLICT OF INTEREST STATEMENT

The authors declare no competing interests.

## References

[phy215651-bib-0001] Ahonen, R. E. (1980). Striated muscle ultrastructure in uremic patients and in renal transplant recipients. Acta Neuropathologica, 50, 163–166. 10.1007/BF00692869 6994424

[phy215651-bib-0002] Akchurin, O. , Sureshbabu, A. , Doty, S. B. , Zhu, Y. S. , Patino, E. , Cunningham‐Rundles, S. , Choi, M. E. , Boskey, A. , & Rivella, S. (2016). Lack of hepcidin ameliorates anemia and improves growth in an adenine‐induced mouse model of chronic kidney disease. American Journal of Physiology. Renal Physiology, 311, 877–889. 10.1152/ajprenal.00089.2016 PMC5130453 27440777

[phy215651-bib-0003] Baek, H. S. , Kang, H. G. , Choi, H. J. , Hil, C. , Ha, I. S. , Han, K. H. , Kim, S. H. , Cho, H. Y. , Jil, S. , Park, Y. S. , Lee, J. H. , Lee, J. , Ahn, C. , & Cho, M. H. (2017). Health‐related quality of life of children with pre‐dialysis chronic kidney disease. Pediatric Nephrology, 32, 2097–2105. 10.1007/s00467-017-3721-5 28685173

[phy215651-bib-0004] Becherucci, F. , Roperto, R. M. , Materassi, M. , & Romagnani, P. (2016). Chronic kidney disease in children. Clinical Kidney Journal, 9, 583–591. 10.1093/ckj/sfw047 27478602 PMC4957724

[phy215651-bib-0005] Brizendine, R. K. , Sheehy, G. G. , Alcala, D. B. , Novenschi, S. I. , Baker, J. E. , & Cremo, C. R. (2017). A mixed‐kinetic model describes unloaded velocities of smooth, skeletal, and cardiac muscle myosin filaments in vitro. Science Advances, 3, 1–10. 10.1126/sciadv.aao2267 PMC5733112 29255801

[phy215651-bib-0006] Callahan, D. M. , Tourville, T. W. , Slauterbeck, J. R. , Ades, P. A. , Stevens‐Lapsley, J. , Beynnon, B. D. , & Toth, M. J. (2015). Reduced rate of knee extensor torque development in older adults with knee osteoarthritis is associated with intrinsic muscle contractile deficits. Experimental Gerontology, 72, 16–21. 10.1016/j.exger.2015.08.016 26343257 PMC4654635

[phy215651-bib-0007] de Souza, V. A. , Oliveira, D. , Barbosa, S. R. , Corrêa, J. O. D. A. , Colugnati, F. A. B. , Mansur, H. N. , Fernandes, N. M. D. S. , & Bastos, M. G. (2017). Sarcopenia in patients with chronic kidney disease not yet on dialysis: Analysis of the prevalence and associated factors. PLoS One, 12, e0176230. 10.1371/journal.pone.0176230 28448584 PMC5407780

[phy215651-bib-0008] Diesel, W. , Knight, B. K. , Noakes, T. D. , Swanepoel, C. R. , van Zyl, S. R. , Kaschula, R. O. C. , & Sinclair‐Smith, C. C. (1993). Morphologic features of the myopathy associated with chronic renal failure. American Journal of Kidney Diseases, 22, 677–684. 8238013 10.1016/s0272-6386(12)80430-6

[phy215651-bib-0009] Diesel, W. , Noakes, T. D. , Swanepoel, C. , & Lambert, M. (1990). Isokinetic muscle strength predicts maximum exercise tolerance in renal patients on chronic hemodialysis. American Journal of Kidney Diseases, 16, 109–114. 10.1016/S0272-6386(12)80563-4 2382645

[phy215651-bib-0010] Dutta, S. , & Sengupta, P. (2016). Men and mice: Relating their ages. Life Sciences, 152, 244–248. 10.1016/j.lfs.2015.10.025 26596563

[phy215651-bib-0011] Dziegala, M. , Josiak, K. , Kasztura, M. , Kobak, K. , von Haehling, S. , Banasiak, W. , Anker, S. D. , Ponikowski, P. , & Jankowska, E. (2018). Iron deficiency as energetic insult to skeletal muscle in chronic diseases. Journal of Cachexia, Sarcopenia and Muscle, 9, 802–815. 10.1002/jcsm.12314 30178922 PMC6204587

[phy215651-bib-0012] Fahal, I. H. , Bell, G. M. , Bone, J. M. , & Edwards, R. H. T. (1997). Physiological abnormalities of skeletal muscle in dialysis patients. Nephrology Dialysis Transplantation, 12, 119–127. 10.1093/ndt/12.1.119 9027785

[phy215651-bib-0013] Fenwick, A. J. , Wood, A. M. , & Tanner, B. C. W. (2017). Effects of cross‐bridge compliance on the force‐velocity relationship and muscle power output. PLoS One, 12, e0190335. 10.1371/journal.pone.0190335 29284062 PMC5746261

[phy215651-bib-0014] Finch, C. A. , Miller, L. R. , Inamdar, A. R. , Person, R. , Sierler, K. , & Mackler, B. (1976). Iron deficiency in the rat. Physiological and biochemical studies of muscle dysfunction. The Journal of Clinical Investigation, 58, 447–453. 10.1172/JCI108489 956378 PMC333200

[phy215651-bib-0015] Foster, B. J. , Kalkwarf, H. J. , Shults, J. , Zemel, B. S. , Wetzsteon, R. J. , Thayu, M. , Foerster, D. L. , & Leonard, M. B. (2011). Association of chronic kidney disease with muscle deficits in children. Journal of the American Society of Nephrology, 22, 377–386. 10.1681/ASN.2010060603 21115614 PMC3029910

[phy215651-bib-0016] Galpin, A. J. , Raue, U. , Jemiolo, B. , Trappe, T. A. , Harber, M. P. , Minchev, K. , & Trappe, S. (2012). Human skeletal muscle fiber type specific protein content. Analytical Biochemistry, 425, 175–182. 10.1016/J.AB.2012.03.018 22469996 PMC3358799

[phy215651-bib-0017] Gerson, A. C. , Wentz, A. , Abraham, A. G. , Mendley, S. R. , Hooper, S. R. , Butler, R. W. , Gipson, D. S. , Lande, M. B. , Shinnar, S. , Moxey‐Mims, M. M. , Warady, B. A. , & Furth, S. L. (2010). Health‐related quality of life of children with mild to moderate chronic kidney disease. Pediatrics, 125, e349. 10.1542/peds.2009-0085 20083528 PMC3663134

[phy215651-bib-0018] Guidi, J. , Offidani, E. , Rafanelli, C. , Roncuzzi, R. , Sonino, N. , & Fava, G. A. (2016). The assessment of allostatic overload in patients with congestive heart failure by clinimetric criteria. Stress and Health, 32, 63–69. 10.1002/SMI.2579 24782081

[phy215651-bib-0019] Hiraki, K. , Yasuda, T. , Hotta, C. , Izawa, K. P. , Morio, Y. , Watanabe, S. , Sakurada, T. , Shibagaki, Y. , & Kimura, K. (2013). Decreased physical function in pre‐dialysis patients with chronic kidney disease. Clinical and Experimental Nephrology, 17, 225–231. 10.1007/s10157-012-0681-8 22911116

[phy215651-bib-0020] Hogan, J. , Schneider, M. F. , Pai, R. , Denburg, M. R. , Kogon, A. , Brooks, E. R. , Kaskel, F. J. , Reidy, K. J. , Saland, J. M. , Warady, B. A. , Furth, S. L. , Patzer, R. E. , & Greenbaum, L. A. (2020). Grip strength in children with chronic kidney disease. Pediatric Nephrology, 35, 891–899. 10.1007/s00467-019-04461-x 31932960 PMC7313477

[phy215651-bib-0021] Höök, P. , Sriramoju, V. , & Larsson, L. (2001). Effects of aging on Actin sliding speed on myosin from single skeletal muscle cells of mice, rats, and humans. American Journal of Physiology. Cell Physiology, 280, 782–788. 10.1152/ajpcell.2001.280.4.c782 11245594

[phy215651-bib-0022] Hurria, A. , Jones, L. , & Muss, H. B. (2016). Cancer treatment as an accelerated aging process: Assessment, biomarkers, and interventions. American Society of Clinical Oncology Educational Book, 35, e516–e522. 10.1200/edbk_156160 27249761

[phy215651-bib-0023] John, S. G. , Sigrist, M. K. , Taal, M. W. , & McIntyre, C. W. (2013). Natural history of skeletal muscle mass changes in chronic kidney disease stage 4 and 5 patients: An observational study. PLoS One, 8, e65372. 10.1371/journal.pone.0065372 23741490 PMC3669290

[phy215651-bib-0024] Kawai, M. , & Halvorson, H. R. (1991). Two step mechanism of phosphate release and the mechanism of force generation in chemically skinned fibers of rabbit psoas muscle. Biophysical Journal, 59, 329–342. 10.1016/S0006-3495(91)82227-5 2009356 PMC1281150

[phy215651-bib-0025] Kooman, J. P. , Kotanko, P. , Schols, A. M. W. J. , Shiels, P. G. , & Stenvinkel, P. (2014). Chronic kidney disease and premature ageing. Nature Reviews. Nephrology, 10, 732–742. 10.1038/nrneph.2014.185 25287433

[phy215651-bib-0026] Krishnan, A. V. , & Kiernan, M. C. (2007). Uremic neuropathy: Clinical features and new pathophysiological insights. Muscle & Nerve, 35, 273–290. 10.1002/MUS.20713 17195171

[phy215651-bib-0027] Leal, D. V. , Ferreira, A. , Watson, E. L. , Wilund, K. R. , & Viana, J. L. (2021). Muscle‐bone crosstalk in chronic kidney disease: The potential modulatory effects of exercise. Calcified Tissue International, 108, 461–475. 10.1007/S00223-020-00782-4 33388899

[phy215651-bib-0028] Lewis, M. I. , Fournier, M. , Wang, H. , Storer, T. W. , Casaburi, R. , Cohen, A. H. , & Kopple, J. D. (2012). Metabolic and morphometric profile of muscle fibers in chronic hemodialysis patients. Journal of Applied Physiology, 112, 72–78. 10.1152/japplphysiol.00556.2011 22016372 PMC3290422

[phy215651-bib-0029] Luden, N. , Minchev, K. , Hayes, E. , Louis, E. , Trappe, T. , & Trappe, S. (2008). Human vastus lateralis and soleus muscles display divergent cellular contractile properties. American Journal of Physiology. Regulatory, Integrative and Comparative Physiology, 295, R1593–R1598. 10.1152/AJPREGU.90564.2008 18815206 PMC2584861

[phy215651-bib-0030] Miller, M. S. , Bedrin, N. G. , Ades, P. A. , Palmer, B. M. , & Toth, M. J. (2015). Molecular determinants of force production in human skeletal muscle fibers: Effects of myosin isoform expression and cross‐sectional area. American Journal of Physiology. Cell Physiology, 308, 473–484. 10.1152/ajpcell.00158.2014 PMC4360030 25567808

[phy215651-bib-0031] Miller, M. S. , Bedrin, N. G. , Callahan, D. M. , Previs, M. J. , Jennings, M. E. , Ades, P. A. , Maughan, D. W. , Palmer, B. M. , Toth, M. J., II , Ades, P. A. , Maughan, D. W. , Palmer, B. M. , & Toth, M. J. (2013). Age‐related slowing of myosin Actin cross‐bridge kinetics is sex specific and predicts decrements in whole skeletal muscle performance in humans. Journal of Applied Physiology, 115, 1004–1014. 10.1152/japplphysiol.00563.2013 23887900 PMC3798822

[phy215651-bib-0032] Miller, M. S. , Callahan, D. M. , Tourville, T. W. , Slauterbeck, J. R. , Kaplan, A. , Fiske, B. R. , Savage, P. D. , Ades, P. A. , Beynnon, B. D. , & Toth, M. J. (1985). Moderate‐intensity resistance exercise alters skeletal muscle molecular and cellular structure and function in inactive older adults with knee osteoarthritis. Journal of Applied Physiology, 122(775–787), 2017–2787. 10.1152/japplphysiol.00830.2016 PMC5407204 28082334

[phy215651-bib-0033] Miller, M. S. , & Toth, M. J. (2013). Myofilament protein alterations promote physical disability in aging and disease. Exercise and Sport Sciences Reviews, 41, 93–99. 10.1097/JES.0b013e31828bbcd8 23392279 PMC4171103

[phy215651-bib-0034] Miller, M. S. , VanBuren, P. , LeWinter, M. M. , Braddock, J. M. , Ades, P. A. , Maughan, D. W. , Palmer, B. M. , & Toth, M. J. (2010). Chronic heart failure decreases cross‐bridge kinetics in single skeletal muscle fibres from humans. Journal of Physiology, 588, 4039–4053. 10.1113/jphysiol.2010.191957 20724360 PMC3000591

[phy215651-bib-0035] Miller, M. S. , VanBuren, P. , LeWinter, M. M. , Lecker, S. H. , Selby, D. E. , Palmer, B. M. , Maughan, D. W. , Ades, P. A. , & Toth, M. J. (2009). Mechanisms underlying skeletal muscle weakness in human heart failure: Alterations in single fiber myosin protein content and function. Circulation. Heart Failure, 2, 700–706. 10.1161/CIRCHEARTFAILURE.109.876433 19919996 PMC2782533

[phy215651-bib-0036] Mitrou, G. I. , Sakkas, G. K. , Poulianiti, K. P. , Karioti, A. , Tepetes, K. , Christodoulidis, G. , Giakas, G. , Stefanidis, I. , Geeves, M. A. , Koutedakis, Y. , & Karatzaferi, C. (2019). Evidence of functional deficits at the single muscle fiber level in experimentally‐induced renal insufficiency. Journal of Biomechanics, 82, 259–265. 30447801 10.1016/j.jbiomech.2018.10.035

[phy215651-bib-0037] Momb, B. A. , Patino, E. , Akchurin, O. M. , & Miller, M. S. (2022). Iron supplementation improves skeletal muscle contractile properties in mice with chronic kidney disease. Kidney360, 3, 843–858. 10.34067/KID.0004412021 36128477 PMC9438424

[phy215651-bib-0038] Mulieri, L. A. , Barnes, W. , Leavitt, B. J. , Ittleman, F. P. , LeWinter, M. M. , Alpert, N. R. , & Maughan, D. W. (2002). Alterations of myocardial dynamic stiffness implicating abnormal crossbridge function in human mitral regurgitation heart failure. Circulation Research, 90, 66–72. 10.1161/hh0102.103221 11786520

[phy215651-bib-0039] Palmer, B. M. , Suzuki, T. , Wang, Y. , Barnes, W. D. , Miller, M. S. , & Maughan, D. W. (2007). Two‐state model of acto‐myosin attachment‐detachment predicts C‐process of sinusoidal analysis. Biophysical Journal, 93, 760–769. 10.1529/biophysj.106.101626 17496022 PMC1913148

[phy215651-bib-0040] Patino, E. , Doty, S. B. , Bhatia, D. , Meza, K. , Zhu, Y. S. , Rivella, S. , Choi, M. E. , & Akchurin, O. (2020). Carbonyl iron and iron dextran therapies cause adverse effects on bone health in juveniles with chronic kidney disease. Kidney International, 98, 1210–1224. 10.1016/j.kint.2020.05.043 32574618 PMC7606334

[phy215651-bib-0041] Peng, H. , Cao, J. , Yu, R. , Danesh, F. , Wang, Y. , Mitch, W. E. , Xu, J. , & Hu, Z. (2016). CKD stimulates muscle protein loss via rho‐associated protein kinase 1 activation. Journal of the American Society of Nephrology, 27, 509–519. 10.1681/ASN.2014121208 26054539 PMC4731120

[phy215651-bib-0042] Petersen, A. C. , Leikis, M. J. , McMahon, L. P. , Kent, A. B. , Murphy, K. T. , Gong, X. , & McKenna, M. J. (2012). Impaired exercise performance and muscle Na(+),K(+)‐pump activity in renal transplantation and haemodialysis patients. Nephrology, Dialysis, Transplantation, 27, 2036–2043. 10.1093/NDT/GFR586 22049181

[phy215651-bib-0043] Pim van der, H. , Rudolf de, B. , Nilesh, S. , Liza, W. , Jardi, H. , Veryan, C. , Hans, H. , Adriaan, V. , Wiek van, G. , Tiny, J. , & Dirk van, V. (2010). Telomere length and outcome in heart failure. Annals of Medicine, 42, 13–18. 10.3109/07853890903321567 19941413

[phy215651-bib-0044] Saydah, S. H. , Xie, H. , Imperatore, G. , Burrows, N. R. , & Pavkov, M. E. (2018). Trends in albuminuria and GFR among adolescents in the United States, 1988‐2014. American Journal of Kidney Diseases, 72, 644–652. 10.1053/j.ajkd.2018.04.021 30017204

[phy215651-bib-0045] Schneider, C. A. , Rasband, W. S. , & Eliceiri, K. W. (2012). NIH image to ImageJ: 25 years of image analysis. Nature Methods, 9, 671–675. 10.1038/nmeth.2089 22930834 PMC5554542

[phy215651-bib-0046] Sethi, S. , D'Agati, V. D. , Nast, C. C. , Fogo, A. B. , de Vriese, A. S. , Markowitz, G. S. , Glassock, R. J. , Fervenza, F. C. , Seshan, S. V. , Rule, A. , Racusen, L. C. , Radhakrishnan, J. , Winearls, C. G. , Appel, G. B. , Bajema, I. M. , Chang, A. , Colvin, R. B. , Cook, H. T. , Hariharan, S. , … Haas, M. (2017). A proposal for standardized grading of chronic changes in native kidney biopsy specimens. Kidney International, 91, 787–789. 10.1016/J.KINT.2017.01.002 28314581

[phy215651-bib-0047] Sgambat, K. , Matheson, M. , Hooper, S. , Warady, B. , Furth, S. , & Moudgil, A. (2019). Prevalence and outcomes of fragility: A frailty‐inflammation phenotype in children with chronic kidney disease. Pediatric Nephrology, 34, 2563–2569. 10.1007/s00467-019-04313-8.Prevalence 31375914 PMC6819247

[phy215651-bib-0048] Slinker, B. K. , & Glantz, S. A. (1985). Multiple regression for physiological data analysis: The problem of multicollinearity. American Journal of Physiology. Regulatory, Integrative and Comparative Physiology, 18, R1–R12. 10.1152/ajpregu.1985.249.1.r1 4014489

[phy215651-bib-0049] Solagna, F. , Tezze, C. , Lindenmeyer, M. T. , Lu, S. , Wu, G. , Liu, S. , Zhao, Y. , Mitchell, R. , Meyer, C. , Omairi, S. , Kilic, T. , Paolini, A. , Ritvos, O. , Pasternack, A. , Matsakas, A. , Kylies, D. , Zur Wiesch, J. S. , Turner, J. E. , Wanner, N. , … Huber, T. B. (2021). Pro‐cachectic factors link experimental and human chronic kidney disease to skeletal muscle wasting programs. Journal of Clinical Investigation, 131, e135821. 10.1172/JCI135821 34060483 PMC8159690

[phy215651-bib-0050] Tanner, B. C. W. , McNabb, M. , Palmer, B. M. , Toth, M. J. , & Miller, M. S. (2014). Random myosin loss along thick‐filaments increases myosin attachment time and the proportion of bound myosin heads to mitigate force decline in skeletal muscle. Archives of Biochemistry and Biophysics, 552–553, 117–127. 10.1016/j.abb.2014.01.015 PMC4043927 24486373

[phy215651-bib-0051] Thurlow, J. S. , Joshi, M. , Yan, G. , Norris, K. C. , Agodoa, L. Y. , Yuan, C. M. , & Nee, R. (2021). Global epidemiology of end‐stage kidney disease and disparities in kidney replacement therapy. American Journal of Nephrology, 52, 98–107. 10.1159/000514550 33752206 PMC8057343

[phy215651-bib-0052] Toth, M. J. , Miller, M. S. , Callahan, D. M. , Sweeny, A. P. , Nunez, I. , Grunberg, S. M. , Der‐Torossian, H. , Couch, M. E. , & Dittus, K. (2013). Molecular mechanisms underlying skeletal muscle weakness in human cancer: Reduced myosin‐Actin cross‐bridge formation and kinetics. Journal of Applied Physiology, 114, 858–868. 10.1152/japplphysiol.01474.2012 23412895 PMC3633441

[phy215651-bib-0053] Webster, A. C. , Nagler, E. V. , Morton, R. L. , & Masson, P. (2017). Chronic kidney disease. The Lancet, 389, 1238–1252. 10.1016/S0140-6736(16)32064-5 27887750

[phy215651-bib-0054] White, R. B. , Biérinx, A. S. , Gnocchi, V. F. , & Zammit, P. S. (2010). Dynamics of muscle fibre growth during postnatal mouse development. BMC Developmental Biology, 10, 21. 10.1186/1471-213X-10-21 20175910 PMC2836990

[phy215651-bib-0055] Zhang, L. , Chen, Q. , Chen, Z. , Wang, Y. , Gamboa, J. L. , Ikizler, T. A. , Garibotto, G. , & Mitch, W. E. (2020). Mechanisms regulating muscle protein synthesis in chronic kidney disease. Journal of the American Society of Nephrology, 31, 2573–2587. 10.1681/ASN.2019121277 32764136 PMC7608956

[phy215651-bib-0056] Zhao, Y. , & Kawai, M. (1993). The effect of the lattice spacing change on cross‐bridge kinetics in chemically skinned rabbit psoas muscle fibers. II. Elementary steps affected by the spacing change. Biophysical Journal, 64, 197–210. 10.1016/S0006-3495(93)81357-2 7679297 PMC1262317

[phy215651-bib-0057] Zhao, Y. , & Kawai, M. (1994). Kinetic and thermodynamic studies of the cross‐bridge cycle in rabbit psoas muscle fibers. Biophysical Journal, 67, 1655–1668. 10.1016/S0006-3495(94)80638-1 7819497 PMC1225527

[phy215651-bib-0058] Zhou, Y. , Hellberg, M. , Svensson, P. , Höglund, P. , & Clyne, N. (2018). Sarcopenia and relationships between muscle mass, measured glomerular filtration rate and physical function in patients with chronic kidney disease stages 3‐5. Nephrology, Dialysis, Transplantation, 33, 342–348. 10.1093/ndt/gfw466 28340152

